# Crystallization and X-ray diffraction analysis of the CH domain of the cotton kinesin GhKCH2

**DOI:** 10.1107/S2053230X16001825

**Published:** 2016-02-19

**Authors:** Xinghua Qin, Ziwei Chen, Ping Li, Guoqin Liu

**Affiliations:** a College of Biological Sciences, China Agricultural University, No. 2 Yuanmingyuanxilu, Haidian District, Beijing 100094, People’s Republic of China; b School of Aerospace Medicine, The Fourth Military Medical University, No. 169 Changlexi Road, Xincheng District, Xi’an 710032, People’s Republic of China

**Keywords:** plant-specific kinesin, CH domain, cotton kinesin GhKCH2

## Abstract

GhKCH2 belongs to a group of plant-specific kinesins (KCHs) containing an actin-binding calponin homology (CH) domain in the N-terminus. Previous studies revealed that the GhKCH2 CH domain (GhKCH2-CH) had a higher affinity for F-actin (*K*_d_ = 0.42 ± 0.02 µ*M*) than most other CH-domain-containing proteins. To understand the underlying mechanism, prokaryotically expressed GhKCH2-CH (amino acids 30–166) was purified and crystallized. Crystals were grown by the sitting-drop vapour-diffusion method using 0.1 *M* Tris–HCl pH 7.0, 20%(*w*/*v*) PEG 8000 as a precipitant. The crystals diffracted to a resolution of 2.5 Å and belonged to space group *P*2_1_, with unit-cell parameters *a* = 41.57, *b* = 81.92, *c* = 83.00 Å, α = 90.00, β = 97.31, γ = 90.00°. Four molecules were found in the asymmetric unit with a Matthews coefficient of 2.22 Å^3^ Da^−1^, corresponding to a solvent content of 44.8%.

## Abstract 2

The cloning, expression, purification and crystallization of the CH domain of the plant-specific kinesin GhKCH2 is reported.

## 1. Introduction   

Microfilaments and microtubules, two vital cytoskeleton systems in cells, together take part in a variety of cellular activities, such as cell division and proliferation, transportation of organelles and vesicles, and the organization and formation of plant preprophase bands, phragmoplasts and cell plates (Wasteneys & Galway, 2003 ▸; Petrášek & Schwarzerová, 2009 ▸). The kinesins are a superfamily of microtubule-based motor proteins (Howard, 1996 ▸), some of which (KCHs) contain a single N-terminal calponin homology (CH) domain that is able to bind to both microfilaments and microtubules. In 2004, the first KCH (GhKCH1) was identified and demonstrated to be involved in the elongation of cotton fibres (Preuss *et al.*, 2004 ▸). Subsequently, our laboratory and others identified further KCHs (GhKCH2, O12/OsKCH1, AtKinG and NtKCH1), all of which were able to bind to both microfilaments and microtubules *in vitro* or *in vivo* (Frey *et al.*, 2009 ▸; Xu *et al.*, 2009 ▸; Buschmann *et al.*, 2011 ▸; Umezu *et al.*, 2011 ▸; Klotz & Nick, 2012 ▸). Recently, Ram Dixit deduced that KCH might be involved in the transportation of actin fragments (Dixit, 2012 ▸).

The actin-binding CH domain, named after its first identification in calponin, consists of about 100 amino-acid residues (Takahashi & Nadal-Ginard, 1991 ▸). Since the first crystal structure of the CH domain was published in 1997 (spectrin from *Homo sapiens*; PDB entry 1aa2; Djinovic Carugo *et al.*, 1997 ▸), along with the first NMR structure published in 2002 (calponin from *Gallus gallus*; PDB entry 1h67; Bramham *et al.*, 2002 ▸), increasing numbers of structures of CH domains from yeast, animals and humans have been solved and deposited in the PDB (http://www.rcsb.org). In comparison, knowledge of plant CH-domain crystal structures remains limited, with only one structure available (fimbrin from *Arabidopsis thaliana*; PDB entry 1pxy; Klein *et al.*, 2004 ▸).

GhKCH2 (GenBank accession No. EF432568), a KCH previously cloned from cotton (*Gossypium hirsutum*) fibres in our laboratory, has a motor domain with microtubule-stimulated ATPase activity and a CH domain that strongly interacts with microfilaments, suggesting it to be a candidate for a linker between microfilaments and microtubules (Xu *et al.*, 2007 ▸, 2009 ▸). The CH domain of GhKCH2 shares 31% amino-acid sequence identity with human calponin 1 and 28% amino-acid sequence identity with *Arabidopsis* fimbrin. Our previous studies revealed that GhKCH2-N (amino acids 1–306), containing the CH domain, had a higher affinity for F-actin (*K*_d_ = 0.42 ± 0.02 µ*M*) than most other CH-domain-containing proteins (*K*_d_ = ∼5–50 µ*M*) (Gimona *et al.*, 2002 ▸; Xu *et al.*, 2009 ▸). To elucidate the mechanisms of this unique biochemical feature, further exploration of the structure of GhKCH2-CH was performed. Here, the expression, purification, crystallization and preliminary X-ray diffraction studies of the CH domain of GhKCH2 are described.

## 2. Materials and methods   

### 2.1. Protein expression and purification   

**Figure 1:**
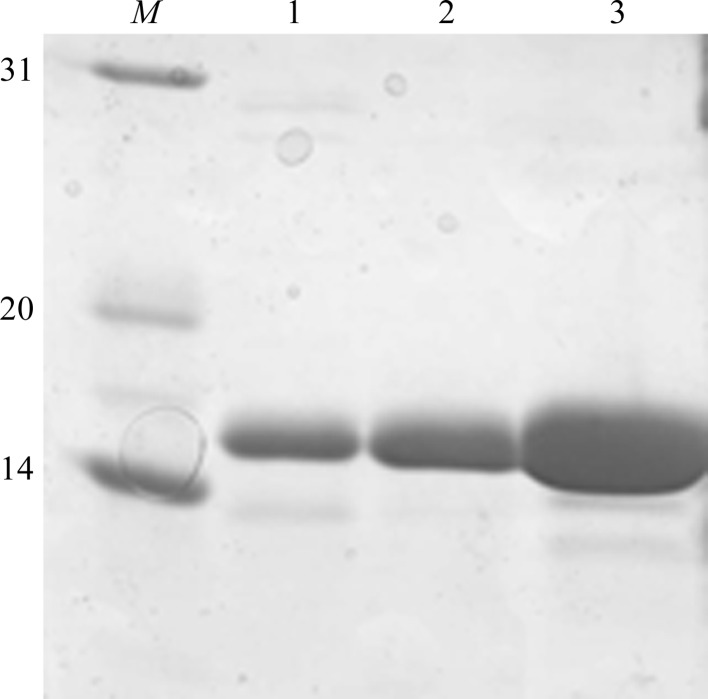
SDS–PAGE analysis of purified GhKCH2-CH. Lane *M*, molecular-weight standards (labelled in kDa); lane 1, purified GhKCH2-CH after GST affinity purification; lane 2, after Mono Q chromatography; lane 3, after gel-filtration chromatography.

The CH domain of GhKCH2 was cloned using the forward primer 5′-GAG AGT C**CA TAT G**GA TTT GGA ATC TAG AAA AGC TG-3′ (NdeI site in bold) and the reverse primer 5′-CAA **CTC GAG** TTA CGA GAG CCT CCA CTC GTT ATA G-3′ (XhoI site in bold). The PCR product was digested and inserted into a modified pGEX-4T-2 vector (kindly provided by Professor Zhongzhou Chen, China Agricultural University) at the NdeI and XhoI restriction sites with a TEV protease cleavage site between GST and the target gene (Table 1 ▸). The correct certified constructs were transformed into *Escherichia coli* strain BL21(DE3). Cells were grown at 37°C in LB medium containing 100 mg ml^−1^ ampicillin to an *A*_600_ of 0.6–0.8 and were induced with 0.1 m*M* isopropyl β-d-1-thiogalactopyranoside (IPTG) at 22°C overnight. The cells were harvested by centrifugation and lysed by gentle sonication in lysis buffer (0.1 *M* Tris–HCl pH 7.5, 150 m*M* NaCl, 1 m*M* DTT, 1 m*M* PMSF). After high-speed centrifugation, the supernatant was loaded onto a home-made Glutathione Sepharose 4B column (GE Healthcare) and incubated for 1 h. After washing with wash buffer (0.1 *M* Tris–HCl pH 7.5, 150 m*M* NaCl), TEV protease was added to cleave the fusion protein overnight. The collected flowthrough was purified by Mono Q chromatography and further polished by gel filtration on a HiLoad 16/60 Superdex 75 pg column (GE Healthcare). The purified proteins were pooled, concentrated to 10–15 mg ml^−1^, flash-cooled in liquid nitrogen and stored at 80°C. Protein purity and identity were assessed by SDS–PAGE with Coomassie Bright Blue staining (Fig. 1 ▸). All of the purification procedures described above were conducted at 4°C.

### 2.2. Protein crystallization   

Initial screening for crystallization conditions was carried out in 48-well sitting-drop plates using the commercially available kits Crystal Screen, Crystal Screen 2 and Index (Hampton Research, USA) at 4°C. Crystals of GhKCH2-CH were initially grown from a mixture of 1 µl protein solution (10–15 mg ml^−1^ in 20 m*M* Tris–HCl pH 7.5, 150 m*M* NaCl) and 1 µl precipitatant solution equilibrated against 100 µl reservoir solution at 277 K.

**Figure 2:**
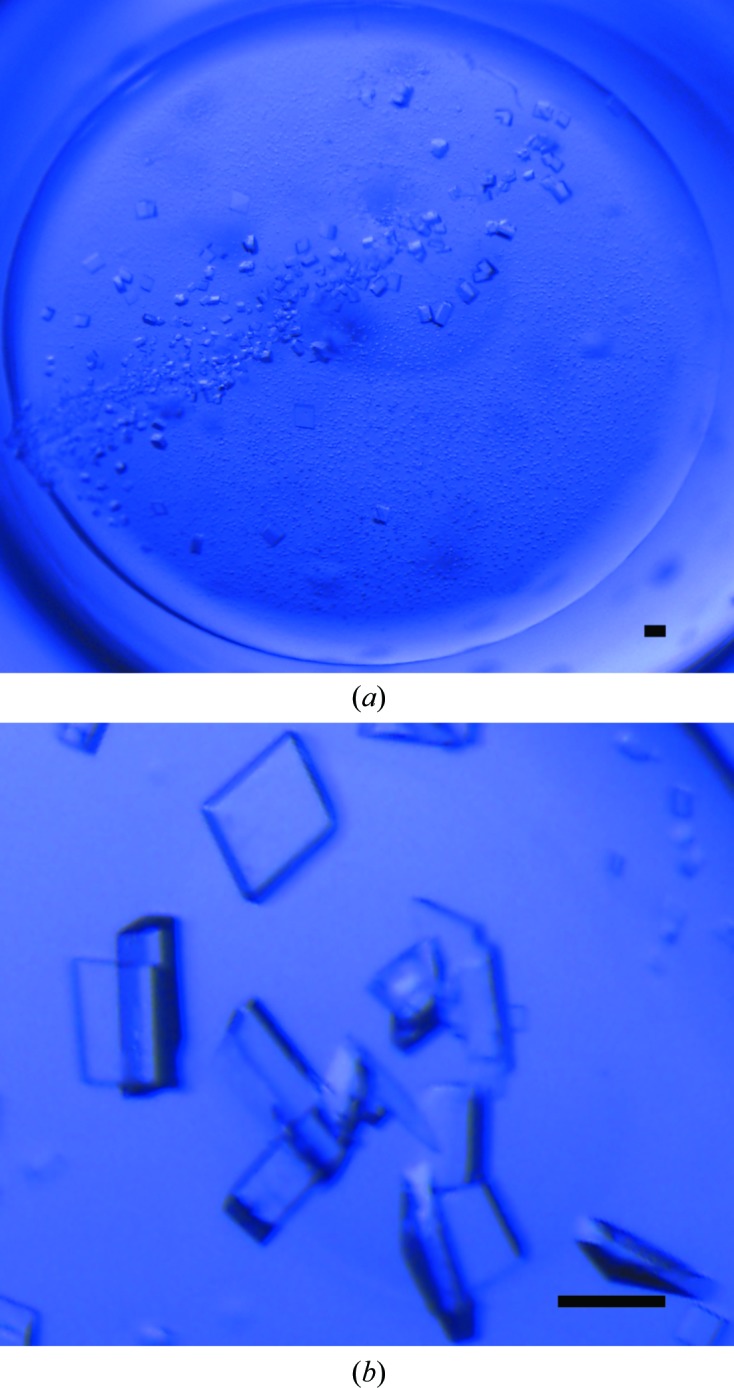
Crystals of GhKCH2-CH. Crystals after streak-seeding (*a*) and enlarged crystals (*b*) are shown. The scale bar is 0.4 mm in length.

Subsequent optimizations were performed using 24-well sitting-drop plates, and the size of the crystals was enlarged by streak-seeding using a cat whisker (Figs. 2 ▸ *a* and 2 ▸ *b*). Detailed information on GhKCH2-CH crystallization is given in Table 2 ▸.

### 2.3. Data collection   

**Figure 3:**
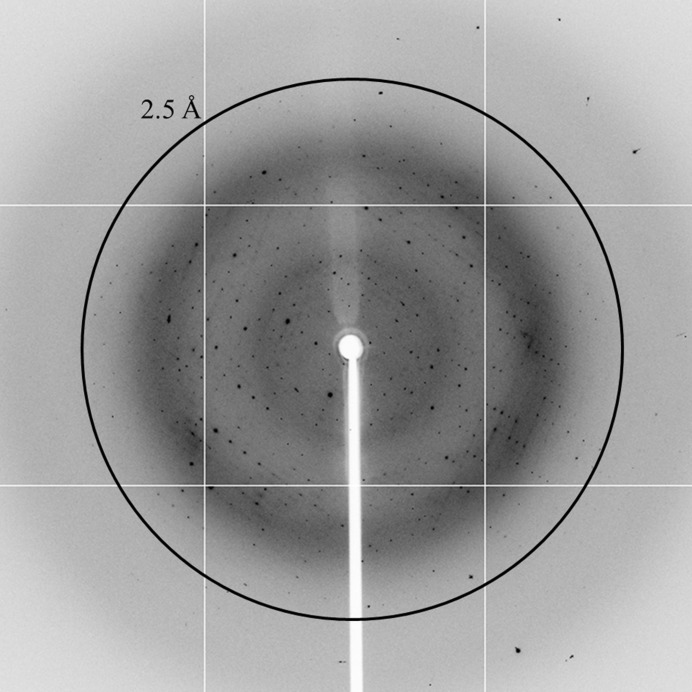
X-ray diffraction pattern from a crystal of GhKCH2-CH. A resolution circle at 2.5 Å is shown.

Mounted crystals were dehydrated with a solution consisting of 0.1 *M* Tris–HCl pH 7.0, 20%(*w*/*v*) PEG 8000, 10%(*v*/*v*) DMSO for 5 min, transferred to a solution consisting of 0.1 *M* Tris–HCl pH 7.0, 20%(*w*/*v*) PEG 8000, 20%(*v*/*v*) DMSO for a further 5 min and finally flash-cooled in liquid nitrogen. The diffraction data set was collected at 100 K on BL17U1 at Shanghai Synchrotron Radiation Facility (SSRF) using an ADSC Q315 CCD. A total of 360 images with an oscillation angle of 1° each were collected using a 250 mm crystal-to-detector distance and an exposure time of 1 s per frame (Fig. 3 ▸). Detailed information on data collection is given in Table 3 ▸.

## 3. Results and discussion   

Indexing with *XDS* (Kabsch, 2010 ▸) indicated that GhKCH2-CH crystallized in space group *P*2_1_. Analysis of the Patterson function with *phenix.xtriage* revealed a significant off-origin peak that was 78.6% of the height of the origin peak (Adams *et al.*, 2010 ▸). Analysis of average intensities with *TRUNCATE* showed that the crystals had strong reflections with indices of *l* = 2*n* [even reflections, *I*/σ(*I*) = 23.6] and weak reflections with indices of *l* = 2*n* + 1 [odd reflections, *I*/σ(*I*) = 13.3] (French & Wilson, 1978 ▸). *HHpred* (http://toolkit.tuebingen.mpg.de/hhpred; Hildebrand *et al.*, 2009 ▸) was used to identify homologues of the GhKCH2 CH domain, and the ten PDB hits (PDB entries 1p2x, 1ujo, 1h67, 1p5s, 1wym, 3i6x, 1wyr, 213g, 1wyp and 1wyn) with the highest scores were used as search models. When determining the crystal structure of GhKCH2-CH using *Phaser* (McCoy *et al.*, 2007 ▸), the structure of human calponin 2 (PDB entry 1wyn; RIKEN Structural Genomics/Proteomics Initiative, unpublished work) gave the best results after further refinement among these models. Four molecules were found in the asymmetric unit with a Matthews coefficient of 2.22 Å^3^ Da^−1^, corresponding to a solvent content of 44.8%.

The entire reflection data set was split into odd (*l* = 2*n* + 1) and even components (*l* = 2*n*) using *MTZUTILS* from the *CCP*4 package (Winn *et al.*, 2011 ▸). The positions of the four molecules in the asymmetric unit were optimized using rigid-body refinement with odd reflections, and restrained refinement was subsequently performed using *REFMAC* or *phenix.refine* with even reflections (Murshudov *et al.*, 2011 ▸; Oksanen *et al.*, 2006 ▸; Adams *et al.*, 2010 ▸). After several cycles of such refinement, the model was refined against all data to an *R*_free_ of about 35%. Owing to the low amino-acid sequence identity of the GhKCH2 CH domain to other CH domain-containing proteins, crystallization of selenomethionine-labelled protein is in progress.

## Figures and Tables

**Table 1:** Macromolecule-production information

Source organism	*G. hirsutum*
DNA source	GenBank accession No. EF432568
Forward primer	5′-GAGAGTCCATATGGATTTGGAATCTAGAAAAGCTG-3′
Reverse primer	5′-CAACTCGAGTTACGAGAGCCTCCACTCGTTATAG-3′
Cloning vector	pGEX-4T-2 (modified)
Expression vector	pGEX-4T-2 (modified)
Expression host	*E. coli* BL21(DE3)
Complete amino-acid sequence of the construct produced	GHMDLESRKAEEDASRRYEAAGWLRKMVGVVAAKDLPAEPSEEEFRLGLRSGIILCNVLNRVQPGAVPKVVESPCDAALIPDGAALSAFQYFENIRNFLVAGQGLGLPTFEASDLEQGGKSARVVNCVLALKSYNEWRLS

**Table 2:** Crystallization

Method	Sitting-drop vapour diffusion
Plate type	24-well sitting drop
Temperature (K)	277
Protein concentration (mg ml^−1^)	10–15
Buffer composition of protein solution	20 m*M* Tris–HCl pH 7.5, 150 m*M* NaCl
Composition of reservoir solution	0.1 *M* Tris–HCl pH 7.0, 20%(*w*/*v*) PEG 8000
Volume and ratio of drop	2 µl, 1:1
Volume of reservoir (µl)	500

**Table 3:** Data collection and processing Values in parentheses are for the outer shell.

Diffraction source	BL17U1, SSRF
Wavelength (Å)	0.9792
Temperature (K)	100
Detector	ADSC Q315 CCD
Crystal-to-detector distance (mm)	250
Rotation range per image (°)	1
Total rotation range (°)	360
Exposure time per image (s)	1
Space group	*P*2_1_
*a*, *b*, *c* (Å)	41.57, 81.92, 83.00
α, β, γ (°)	90.02, 97.31, 90.00
Mosaicity (°)	0.212
Resolution range (Å)	41.23–2.50 (2.65–2.50)
Total No. of reflections	140679 (13539)
No. of unique reflections	19060 (1869)
Completeness (%)	98.82 (97.34)
Multiplicity	7.4 (7.2)
〈*I*/σ(*I*)〉	16.83 (3.83)
*R*_meas_ (%)	7.283 (56.86)
Overall *B* factor from Wilson plot (Å^2^)	52.40
